# Organosilanized Hydrophobic Sand for Drought Resilience: Reducing Water Percolation and Enhancing Crop Growth Conditions

**DOI:** 10.1021/acsomega.5c03952

**Published:** 2025-08-14

**Authors:** Yashwanth Arcot, Ramya Srinivas, Minchen Mu, Mahshad Maghoumi, Luis Cisneros-Zevallos, Mustafa E. S. Akbulut

**Affiliations:** † Artie McFerrin Department of Chemical Engineering, 14736 Texas A&M University, College Station, Texas 77843, United States; ‡ Department of Horticultural Sciences, Texas A&M University, College Station, Texas 77843, United States; § Department of Materials Science and Engineering, Texas A&M University, College Station, Texas 77843, United States

## Abstract

Recently, increasing frequency and severity of drought events have resulted in significant crop yield reductions worldwide, indicating the critical need for innovative agricultural water management strategies to enhance water use efficiency. Addressing this challenge, we present a novel approach involving the strategic placement of highly hydrophobic sand layers below the subrhizosphere. This method utilizes silica sand modified via a facile, single-step surface treatment, yielding a material with strong hydrophobicity, characterized by a static water contact angle of 133.0 ± 1.0°. Importantly, the modified sand demonstrated stability and retained its hydrophobic properties under simulated adverse agricultural conditions. Systematic investigations of the hydraulic properties revealed that the incorporation of these hydrophobic sand layers substantially controlled the vertical infiltration flux of irrigation water. Specifically, a hydrophobic sand layer with an areal density of 796.5 mg/cm^2^ extended the water infiltration time by a factor of approximately 5.5 relative to control soil columns, even following 14 days of sustained irrigation. This engineered impedance promotes saturation within the rhizosphere, thereby potentially enhancing the efficiency of root water uptake. Furthermore, experimental observations indicated a positive correlation between the presence of the hydrophobized subsoil layer and the retention of organic matter within the overlying soil matrix, suggesting ancillary benefits for long-term soil fertility maintenance. Consequently, deploying subrhizosphere hydrophobization using organosilanes as a preplanting soil conditioning treatment presents a potentially more applicable strategy for improving water conservation and soil health, particularly in water-scarce agricultural regions.

## Graphical abstract

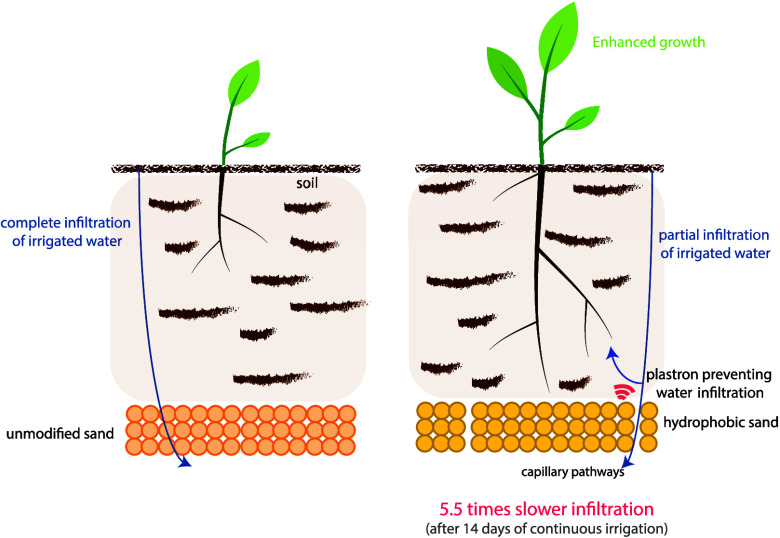

## 1 Introduction

Optimal utilization of available resources while maximizing yield efficiency is a key approach to promoting sustainable agriculture.^1^ In the past decade, many soil amendment strategies have been proposed to promote sustainable agriculture.^2–5^ To optimize the efficacy of soil amendments, it is critical to improve the water-holding capacity of soil.^6^ The seepage of irrigation water from agricultural land poses significant challenges that can undermine water efficiency and result in substantial loss of precious resources.^7,8^

An optimal and efficient irrigation scenario would involve the retention of water within the rhizosphere for extended durations, ensuring its maximum utilization for plant growth.^9^ However, in practice, irrigation frequently results in rapid water percolation beyond the root zone, a phenomenon known as deep percolation, which provides insufficient time for effective water uptake by roots.^10–14^ As this water infiltrates deeper layers, it carries away vital nutrients and agrochemicals from the soil, a process known as leaching.^15^ This not only depletes soil fertility but is a potential environmental concern, contaminating groundwater reserves, giving rise to quality concerns of drinking water and the health of aquatic ecosystems.^16,17^

Water flow dynamics in soil is influenced by several factors, including wetting characteristics, texture, and porosity of the soil.^18^ In sandy soils with hydrophilic tendencies, fine texture, the larger pore sizes result in rapid water infiltration rates but reduced retention.^19^ In silty or clayey soils with high hydrophilicity, granular structure, high porosity, and small pore size distribution, strong capillary forces ensure sustained water retention, albeit with reduced percolation rates and disrupted water flow behavior.^20–22^ Introducing hydrophobicity fundamentally alters pore-scale fluid behavior and macroscopic infiltration dynamics, often leading to the formation of preferential flow paths.^23^ These paths, characterized by fingered or funneled flow, result in serpentine downward water movement, contacting a significant portion of the soil matrix and thereby increasing the water-holding capacity of soil above the hydrophobic zones.^24,25^ On a granular level, the interface in hydrophobic soils is discontinuous, leading to the entrapment of air pockets that delay pore and intergranular space saturation.^26,27^ Introducing hydrophobicity into the deeper layers of the soil would collectively retard the net vertical infiltration flux, thereby increasing water residence time in the subsurface layers. This increased residence time would allow more water to saturate the pores and interstitial gaps between granules, leading to enhanced storage in the unsaturated zone above the hydrophobic region. Soil hydrophobicity can be naturally enhanced by the presence of certain organic compounds, especially long-chain aliphatic acids and hydrocarbons, which favor plant growth.^28,29^ While natural soil hydrophobicity can arise from adsorbed organic compounds, engineered hydrophobicity offers a targeted approach to modify soil hydraulic properties.^30,31^

Previous research has investigated strategies for optimizing irrigated water utilization, including the incorporation of natural mulches as topsoil and the enhancement of sand’s hydrophobic properties through surface modification with paraffin wax.^32–34^ There is a need for facile hydrophobization of the sand bed without relying on pretreated bulk materials, which may cause logistical hurdles for field-scale application. This study investigates a chemical modification strategy (that can potentially be performed in situ): making a subrhizosphere soil layer hydrophobic via organosilanization. Specifically, we utilize octadecyltrichlorosilane (OTS), an organosilane known to react readily with surface hydroxyl groups (−OH) present on silicate minerals abundant in many soils. This reaction covalently grafts hydrophobic octadecyl (−C_18_H_37_) moieties onto the mineral surfaces, drastically increasing the water contact angle and imparting nonwetting characteristics. Notably, OTS is different from persistent PFAS compounds, and related organosilane modifications (PFAS-free) have shown utility in environmental remediation contexts.^35,36^

To date, systematic studies quantifying the impact of facilely generated hydrophobic barriers via organosilanization on agricultural water infiltration kinetics, especially at the subroot soil level, are lacking. This research aims to bridge this gap by investigating how varying degrees of hydrophobicity, achieved through controlled OTS treatment of a sand layer simulating a subsoil horizon, affect water infiltration dynamics over time.

In this investigation, successful chemical modification of sand with an OTS was confirmed using attenuated total reflectance Fourier transform infrared spectroscopy (ATR-FTIR) and by assessing its wetting properties via static contact angle (SCA) measurements using the sessile drop method. To investigate the effectiveness of the hydrophobic sand bed formed by the OTS modification, we created a series of experimental soil systems. In these systems, we systematically varied the OTS-modified sand bed and examined its effectiveness in water retention by observing water infiltration kinetics, total water retention, and the fate of dissolved organic constituents. Furthermore, to preliminarily investigate the impact of hydrophobic sand beds on plant growth and provide direction for future research, plant growth was monitored following the cessation of irrigation, simulating arid conditions. This lab-scale investigation serves as an important step toward developing novel soil amendment strategies. Our goal, through the utilization of hydrophobic sand bed, is to enhance both water retention in arable lands and the prolonged retention of dissolved organic matter, thereby improving crop water availability (demonstrated by schematic; Figure 1). This novel strategy represents a critical foundational step in water conservation and sustainable agriculture.

**Figure 1:**
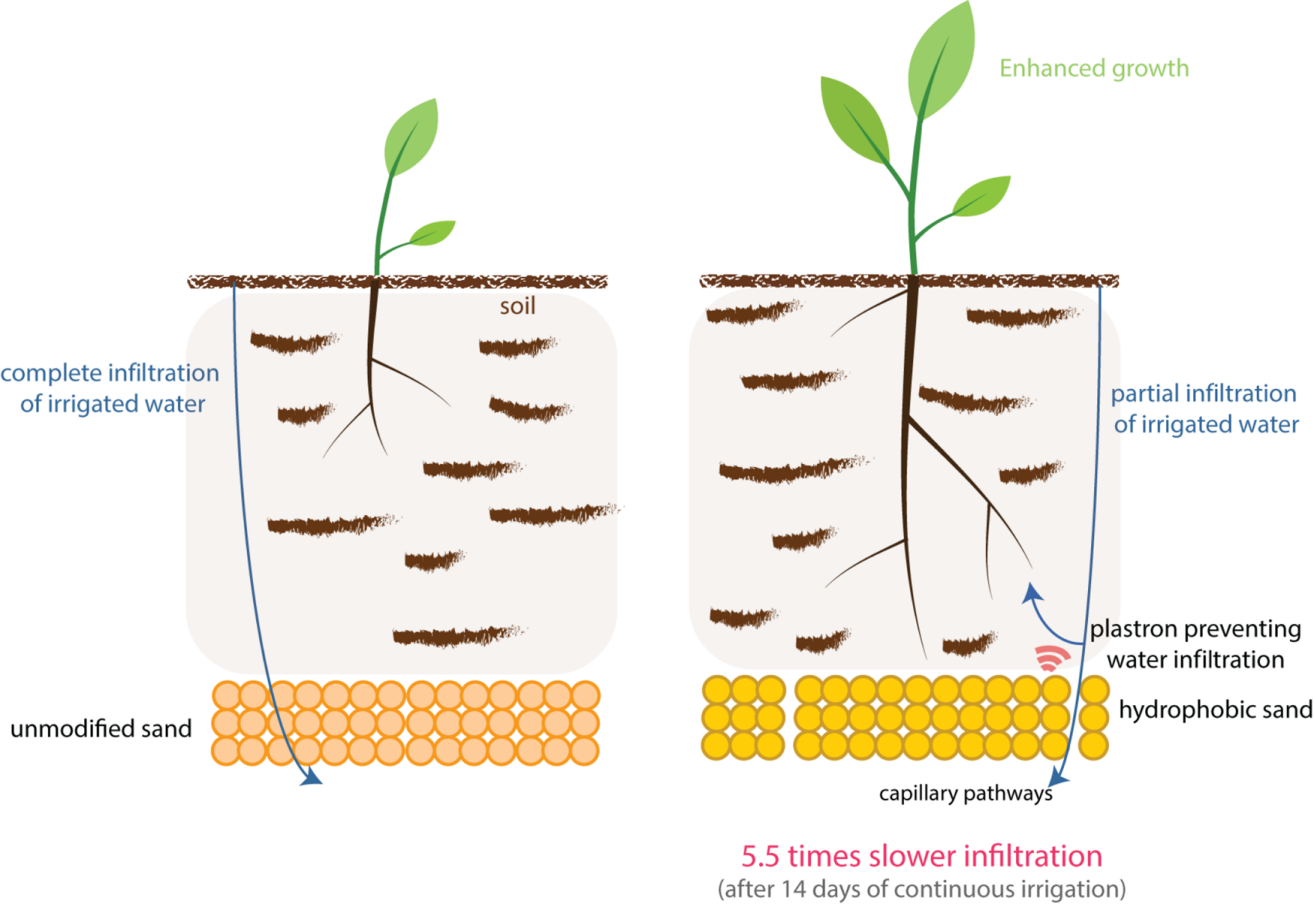
Schematic representation of the experimental setup. The system on the left illustrates an unmodified sand bed, enabling rapid water infiltration. The system on the right demonstrates delayed infiltration caused by the formation of capillary pathways between hydrophobic sand granules. This delay increases the local water potential near the root zone, thereby supporting improved plant growth under arid or water-limited conditions.

## 2 Materials and Methods

### 2.1 Preparation of Hydrophobic Sand

Silica sand (mesh size 40–100, with an average particle diameter of 300 μm) was modified with octadecyltrichlorosilane (OTS, ≥95%, Thermo Fisher Scientific, Waltham, MA) to make it nonwetting/highly hydrophobic. Briefly, a 13.0 mM solution of OTS was prepared in hexane (ACS grade; Avantor Performance Materials, LLC, Center Valley, PA) following our prior studies.^37–39^ 500 g/L suspension of sand (1 g of OTS per 100 g of sand) was then dispersed in the hexane–OTS solution and shaken at 1000 rpm for 24 h. After this period, the hydrophobic sand was allowed to precipitate and the supernatant was discarded. The hydrophobic sand was subsequently washed with fresh hexane to remove any unreacted OTS. Finally, the OTS-modified sand was dried in an oven at an ambient temperature (37 °C) for 12–16 h.

### 2.2 Characterization of Hydrophobic Sand

#### 2.2.1 Chemical Characterization

The modification of the sand with an OTS was confirmed through ATR-FTIR. The FTIR spectra were acquired using a Nicolet iS5 FTIR spectrometer (Thermo Fisher Scientific, Waltham, MA), equipped with a diamond ATR crystal. Spectral analysis was performed using Omnic software (version 9.2.86, Thermo Fisher Scientific). The raw spectral data were extracted and subsequently replotted using OriginPro 2021 for further analysis.

#### 2.2.2 Physical Characterization

The most critical physical characterization of the hydrophobic sand, which is proposed to be incorporated below the soil layer to enhance the water retention capacity (WRC) of the system, is its wettability. Wettability was assessed under various conditions: using deionized (DI) water, water at pH 4.0 (acidic conditions), pH 9.0 (alkaline conditions), water containing dissolved organic constituents on the hydrophobic sand, and static contact angles (SCA) of DI water on sand exposed to sunlight. The pH of the solutions was adjusted to 4 and 9 by adding hydrochloric acid (Fisher Chemical, Waltham, MA) and sodium hydroxide (Sigma-Aldrich, Burlington, MA), respectively, to DI water. The pH was verified by using pH indicator strips (Avantor Performance Materials, LLC, Center Valley, PA). To assess the SCA on hydrophobic sand substrates exposed to sunlight, the modification process followed the procedure described by Bae et al.^40^ Briefly, a UV-AB lamp (160W, REPTI ZOO, Miami, FL) was positioned 15 cm above the hydrophobic sand particles places in a weighing paper, and the samples were irradiated at an intensity of 90–100 W/m^2^, with a temperature of approximately 50 °C for 12 h. After exposure, the SCA was measured using DI water. To measure the SCA using water containing dissolved organic matter from the soil, 3.5 g of soil was added to 10 mL of DI water, simulating the experimental conditions (explained in subsequent sections). The mixture was sonicated for 30 min to allow the organic matter to dissolve into the water, and the supernatant and precipitate were discarded. The SCA was then measured by using the dispersant with dissolved organic matter. All SCA measurements were performed using the sessile drop method, where a 5 μL droplet of the respective water sample was placed on the hydrophobic sand substrate.

A high-resolution camera (E3ISPM Series, ToupTek, China) was employed to capture images of the droplet on the surface, with appropriate settings for brightness, contrast, and white balance to clearly define the liquid–air, liquid–solid, and solid–air interfaces. The SCA were then measured using the Low-Bond Axisymmetric Drop Shape Analysis (LB-ADSA) method, utilizing the Drop Analysis plugin in ImageJ software (version 1.8.0_322 64-bit, National Institute of Health, Bethesda, MD). The above measurement procedure was repeated at least 6 times to ensure reproducibility. To visually demonstrate the contrasting wetting behaviors of unmodified and surface-modified sand, aqueous droplets colored with red (FD&C Red, Sigma-Aldrich, Burlington, MA) and blue (Procion blue, Alfa Aesar, Haverhill, MA) dyes were deposited onto each sand variant. The images were captured using a high-resolution digital camera (Panasonic LUMIX GH5) to demonstrate the differences in wettability.

### 2.3 Evaluation of Hydrophobic Sand: Simulating Agricultural Conditions

#### 2.3.1 Experimental Setup

The experimental setup consisted of six sample conditions, to comprehensively simulate agricultural conditions, where the top layers constitute soil and underlying layers are predominantly sand.^41^ Each sample was named based on the amount of hydrophobic sand added per unit surface area of the container (refer Table 1). It is important to note that the hydrophobic sand was always added to the bottom of the container, making a uniform layer. The names of each sample, the amounts of hydrophobic sand, unmodified sand, and dry soil used (purchased from Jolly Gardener, consisting of 65% Canadian Sphagnum Peat Moss, medium perlite, and vermiculite) are detailed in Table 1.

**Table 1:** Composition of Substrates Used to Simulate Agricultural Soil Conditions, Incorporating Varying Proportions of Hydrophobic and Unmodified Sand in the Bottom and Intermediate Layers

sample name	areal density of hydrophobic sand (in mg/cm^2^) (bottom layer)	areal density of unmodified sand (in mg/cm^2^) (intermediate layer)	amount of dry soil (in g) (top layer)
control	0.00	796.5	35.00
17.70 mg/cm^2^	17.70	778.30	35.00
44.24 mg/cm^2^	44.24	752.22	35.00
88.50 mg/cm^2^	88.50	707.96	35.00
177.00 mg/cm^2^	177.00	619.46	35.00
796.46 mg/cm^2^	796.46	0.00	35.00

Standard 18 oz plastic cups (purchased from a local departmental store) were used as experimental containers. A hole of 1 cm diameter was made at the bottom of each cup to allow water drainage. A cheesecloth, cut into 6 cm × 12 cm dimensions, was folded into half and placed at the bottom of each container to prevent sand loss and only allow for water infiltration. The hydrophobic sand, unmodified sand, and dry soil were added sequentially in this order (with the amounts mentioned in Table 1). We used three replicates for each experimental condition (conditions shown in Table 1) to ensure reproducibility. In all experimental setups, the combined height of the hydrophobic sand bed and unmodified sand column was approximately 1.5 cm, while the soil column was 7 cm tall. Each layer was evenly distributed to ensure uniform coverage of the cheesecloth or the preceding layer (a sample experimental setup is shown in Figure 4). Each sample was irrigated daily with 150 mL of tap water with an approximately consistent irrigation procedure, with a 24 h interval between irrigations. The sample systems were deliberately oversaturated to evaluate the sand bed’s durability under elevated hydrostatic pressure, a condition known to induce air pocket compression and plastron destabilization.^42,43^ The drained water from each sample was collected in a measuring cylinder placed beneath each sample. The entire setup was subjected to a 12 h photoperiod under a fluorescent light setup (Agrobrite, CA). All experiments were conducted under a fluorescent light setup at a constant temperature of 25 °C and a controlled relative humidity of 55 ± 5%.

#### 2.3.2 Water Retention Capacity (WRC)

It was observed that water drainage completely stopped after 1 h of irrigation. Therefore, the volume of water drained was measured 1 h after the addition of 150 mL of tap water to each of the abovementioned samples. The water-holding capacity (WHC) was calculated as the percentage of water retained relative to the weight of the total constituents of the samples (125 g for all of the samples), following the formula reported in previous studies.^44^ WHC measurements were recorded on days 1, 2, 3, 5, 7, and 14 post irrigation for all of the samples.

#### 2.3.3 Water Infiltration Studies

To monitor water infiltration, the flow of water through the hole at the bottom of each sample, simulating the agricultural condition, videos were recorded in real time using a Nikon EOS 70D digital camera (the videos were captured at a resolution of 1920 × 1080 pixels, with a frame rate of 24 frames per second, an aperture setting of f/3.5, shutter speed of 1/30 s, and ISO sensitivity of 800) with consistent camera settings for all samples. Videos were captured during the first 10 min of infiltration. Post irrigation (150 mL of tap water), the volume of infiltrated water was measured in a measuring cylinder positioned beneath each sample at time intervals: 30, 60, 90, 120, 150, 180, 300, 450, and 600 s. This infiltration monitoring procedure was conducted on days 1, 7, and 14 of the experiment.

#### 2.3.4 CIELAB Color Analysis of Drained Water and Soil

To evaluate the subtle color changes in infiltrated water following irrigation and in dried soil samples, colorimetric analysis was performed using the CIELAB color space (L*, a*, b* values) based on the Munsell color system.^45^ The color of the infiltrated water was measured using a CT-310 Chroma Meter (Konica Minolta, Japan) after proper calibration. Water samples were collected post infiltration, transferred to quartz cuvettes, and analyzed for colorimetric parameters.

To analyze the color variations in the soil samples, the soil samples were first vacuum-dried at 35 °C for 24 h. The dried soil was then sieved through a mesh size of 35 (pore diameter: 500 μm), and the collected fraction was uniformly spread on a white background for analysis. A Chroma Meter CR-310 (Konica Minolta, Japan), equipped with a xenon arc lamp light source and a 50 mm diameter measurement area, was calibrated and employed to measure the soil color parameters.

Color measurements for both water and soil samples were conducted on days 1, 7, and 14 of the experiment under consistent environmental and instrumental conditions. From the obtained a* and b* values, two colorimetric parameters (hue angle and chroma) were calculated, and L*, hue angle, and chroma values were reported.^46,47^

To quantify the change in dissolved organic constituents within the infiltrated water, UV–vis spectroscopy (UV-1800, Shimadzu Corp., Columbia, MD) was employed. The dissolved organic matter exhibited a maximum absorbance at 287 nm, consistent with previously reported UV absorption characteristics.^48^ 1 mL aliquots of the infiltrate were diluted with 9 mL of ethanol. Subsequently, the percentage decrease in dissolved organic matter was calculated utilizing the absorbance value at 287 nm (as absorbance and the concentration of dissolved organic matter exhibit a linear relationship, in accordance with the Beer–Lambert Law), by comparing samples from varying hydrophobic sand bed configurations to control samples on day 7 of the infiltration studies.^49^

### 2.4 Impact of Hydrophobic Sand on Plant Growth

Preliminary experiments were conducted to investigate the effect of the hydrophobic sand bed on plant growth. Two model growth conditions were established using acrylic plastic containers (8″ × 6″ × 2″). To facilitate drainage, three holes were incorporated into the base of each container. A cheesecloth layer was introduced above the drainage holes to prevent the loss of sand. Each container was filled with a thick layer (∼3000 mg/cm^2^) of either hydrophobic or unmodified sand followed by equal amounts of moist soil. Tomato (tomate grande rojo, USDA, Norton, MA) seeds were sown in each container at a density of eight seeds per container. A controlled irrigation protocol was implemented, delivering equal volumes of water (∼150 mL) to each container. It is important to note that this protocol differs from the previously described experimental setup in one critical aspect, i.e, the irrigation of the soil systems. Irrigation was performed daily to maintain a consistent soil moisture (as in the previous experimental design). Throughout the 14-day experimental period, samples were maintained under a 12 h photoperiod (utilizing the light source as mentioned previously). Irrigation was terminated on day 14, while the 12 h photoperiod was continued for an additional 10 days. The growth of tomato seedlings was monitored during these 10 days postirrigation seizure.

### 2.5 Statistical Analysis

Since each experimental condition included at least three replicates, the mean and standard error were calculated and reported for all measured parameters. For all experimental and characterization assays (except for ATR-FTIR micrographs), data are presented as the mean ± standard error. To evaluate the statistical significance among different sample conditions, one-way analysis of variance (ANOVA) was performed for SCA, WHC, and colorimetric parameters (L*, hue angle, and chroma) across all six sample groups on days 1–14. Tukey’s posthoc test was conducted with a 95% confidence interval to determine statistical distinction. Statistically distinct groups were indicated using different letters (A, B, C, D).

## 3 Results and Discussion

### 3.1 Characterization of Hydrophobic Sand

ATR-FTIR spectroscopy was performed to confirm that the surface modification of sand was successful and via covalent bond formation to ensure chemical stability and functional performance. Upon observing the complete ATR-FTIR spectrum (ranging from 4000 to 400 cm^–1^), two distinct peaks were observed at 2917 and 2846 cm^–1^ in the spectrogram of the silane and hydrophobic sand, but absent in the unmodified sand (represented as unmodified sand in the spectrograms), corresponding to the C–H stretching vibrations of CH_2_ and CH_3_ moieties (shown in Figure S1). This observation aligns with previous findings and the inherent structure of sand, which lacks CH_2_ and CH_3_ functional groups.^50^ A sharp peak at 1079 cm^–1^, attributed to the Si–O–Si stretching vibrations, was initially observed in the sand spectra^51^ (Figure 2). Upon reaction with the OTS, a blue shift of 5 cm^–1^ was observed in this Si–O–Si peak (new Si–O–Si peak observed at 1083 cm^–1^), indicating a reduction in bond length within the newly formed Si–O–Si bonds. This shift signifies the formation of stronger bonds between the Si–O–Si functional groups present in the sand and the Si–Cl_*x*_ moieties within the OTS. Additionally, a peak with low intensity was detected in the spectra of the OTS-modified sand particles at 578 cm^–1^, which was absent in the spectrograms of the sand.^52^ This observed peak corresponds to the Si–Cl_*x*_ stretching vibration, further confirming successful chemical modification of the sand surface.

**Figure 2:**
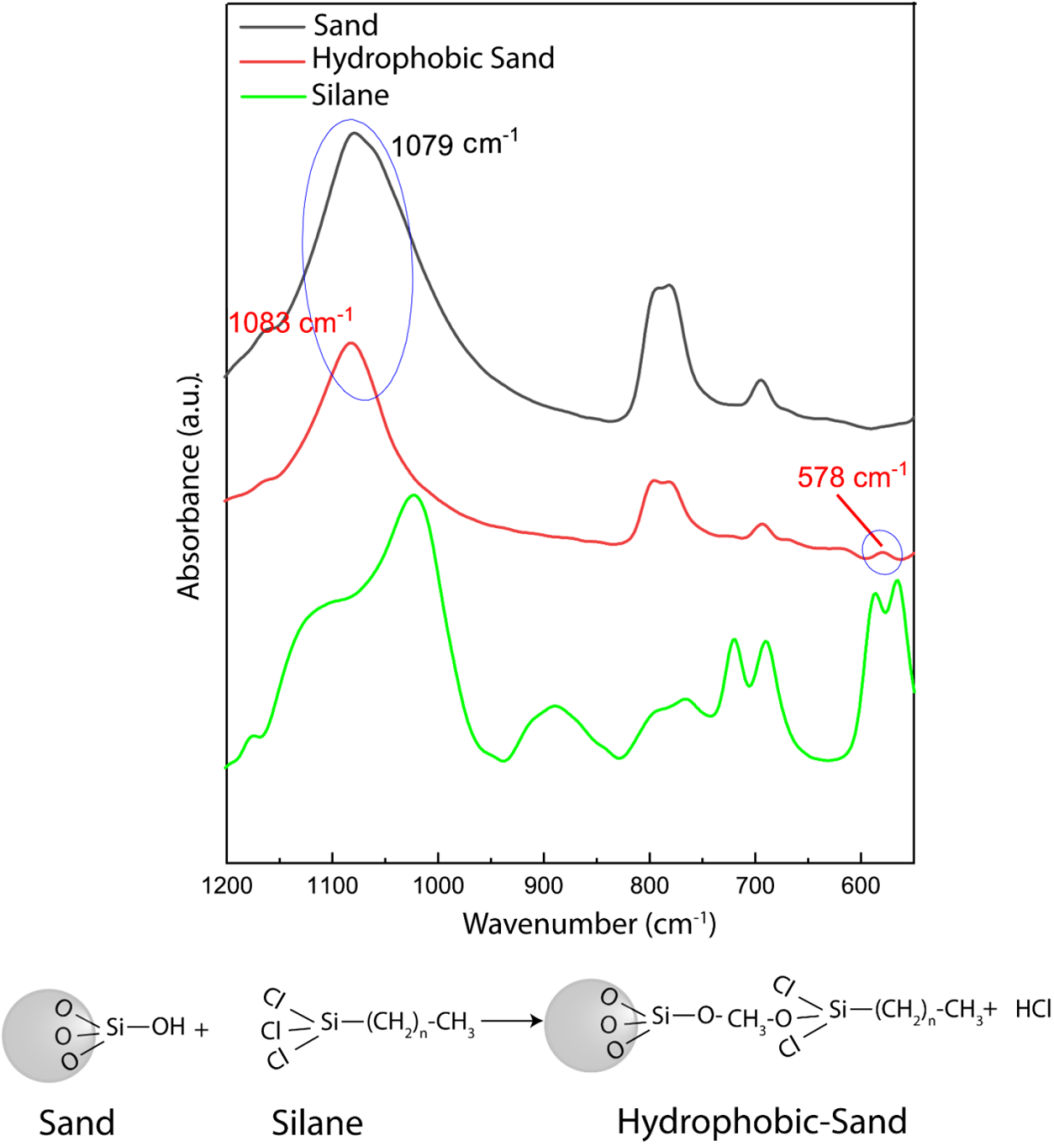
ATR-FTIR spectra (1200–500 cm^–1^) illustrating the surface functionalization of silica sand. Spectra comparing surface functional groups on unmodified sand, octadecyltrichlorosilane, and octadecyltrichlorosilane-modified hydrophobic sand. The chemical schematic (shown below the spectrogram) depicts the proposed surface functionalization of sand.

Delayed water infiltration can be achieved by introducing metastable trapped air at the bottom layer of the soil profile, which typically consists of sand in agricultural soils.^41^ This can be done by altering the capillary force dynamics during water flow through the introduction of trapped air, which can impede the downward movement of water, thereby enhancing water retention in the upper soil layers. For this, the nonwetting properties of the hydrophobic sand were characterized at various conditions (shown in Figure 3). SCA measurements of DI water on the hydrophobic sand surface showed a value of 133.0 ± 1.0°. The addition of acid (to adjust pH to 4.0), base (to adjust pH to 9.0), and dissolved organic matter resulted in a statistically significant reduction in SCA. However, the values remained relatively high, ranging between 123.2 ± 0.2 and 127.1 ± 1.2°. The optimal soil pH range for the growth of various plant species typically lies between 5.5 and 8.0.^53^ Therefore, pH values of 4.0 and 9.0 were selected to represent the extreme conditions outside this range. Furthermore, the hydrophobic sand was subjected to UV-AB irradiation for 12 h to simulate harsh sunlight conditions and assess potential photodegradation. The hydrophobic sand particles were exposed to an irradiation intensity of 90–100 W/m^2^, which is significantly higher than the average solar irradiance received by typical croplands, approximately 28 W/m^2^.^54^ The sunlight exposure resulted in a 5° decrease in the SCA.

**Figure 3:**
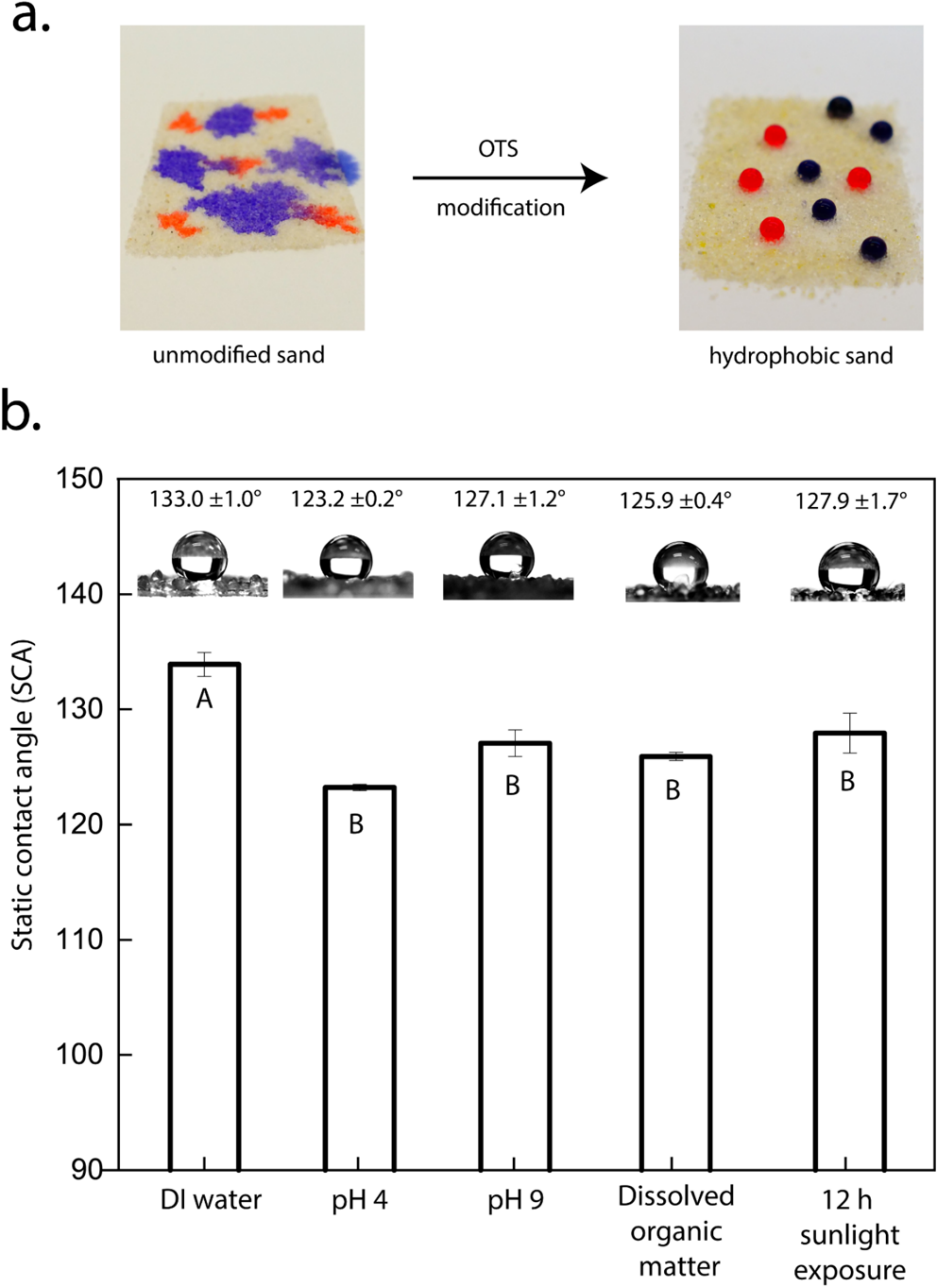
(a) High-resolution images showing the wetting behavior of unmodified and hydrophobic sand using colored water (DI water dyed with FD&C Red and Procion Blue). (b) Graph represents the static contact angle values (measured by the sessile drop method) using water droplets on hydrophobic sand under the following conditions: DI water, water adjusted to pH 4, water adjusted to pH 9, and water with dissolved organic matter, contact angles of DI water on hydrophobic sand samples exposed to 12 h of sunlight. Contact angles noted with different letters indicate statistically significant differences (*p* < 0.05).

Since all measured SCA values exceeded 120°, which is generally considered the upper limit for complete wetting in the Wenzel state, all of the SCAs measured including DI water, water at two selected pH values (4.0 and 9.0), and water containing dissolved organic matter, as well as SCA measured on hydrophobic sand following UV exposure, exhibited characteristics falling within the Cassie–Baxter wetting regime. This behavior indicates the presence of metastable air pockets entrapped within the interstitial spaces between the sand granules.^55^

### 3.2 Water Retention Capacity of Soil Systems

The experimental design in this study involved studying water retentions in oversaturated model agricultural samples. This approach was deliberately chosen to evaluate the durability of the plastron: the entrapped air layer within the interstitial spaces and between the layers of the hydrophobic sand under excessive hydraulic head.^56^ In the control sample, on day 1, approximately 44.80% of the 150 mL of water added was retained by the dried soil and unmodified sand (refer to Figure 4). While on day 1, the WRC of the samples containing 17.70, 44.24, 88.50, and 177.00 mg/cm^2^ of hydrophobic sand exhibited a positive correlation with the surface coverage of hydrophobic sand applied, indicating that increased loading of the hydrophobic layer enhances the initial water retention. Above an areal density of 88.50 mg/cm^2^, of the hydrophobic sand, complete inhibition of infiltration was observed. This phenomenon can be attributed to the enhanced thermodynamic stability of the plastron. As mentioned by Marmur et al., the underwater–water repellency is fundamentally governed by parameters including surface roughness (*r*), the solid–liquid contact area (φ_s_), and wetting properties of the smooth solid substrate (θ).^57^ Critically, the roughness of the hydrophobic sand and the resultant solid–liquid contact area directly influence the volume and stability of the entrapped plastron. Consistent with the Cassie–Baxter model and previous literature, increased volume of trapped air (positively correlated with surface roughness at the nano/microscale) thermodynamically favors a reduction in the solid–liquid interface, thereby impeding water infiltration.^58^

**Figure 4:**
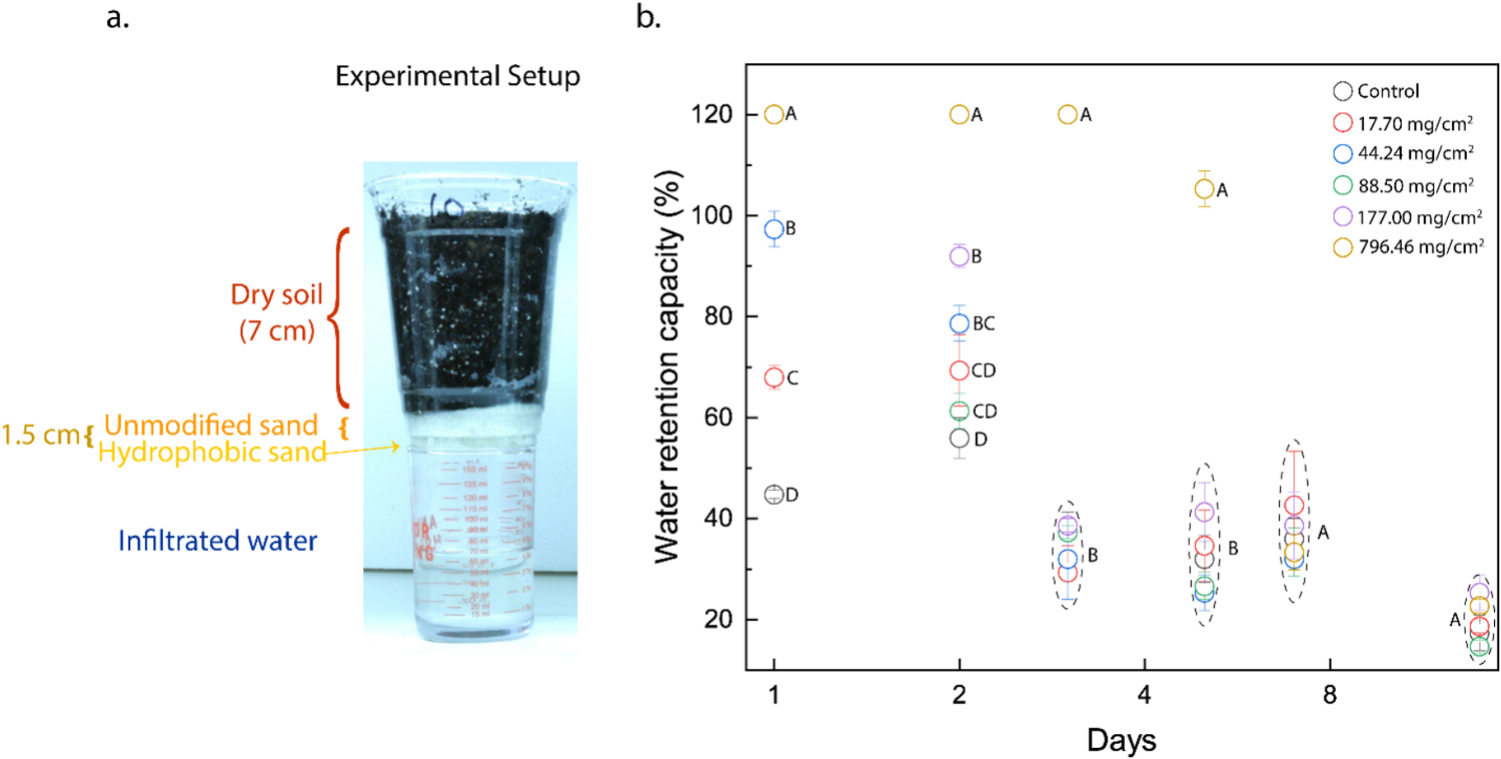
(a) Digital image showing the experimental setup for water retention and infiltration studies. The setup comprises a top layer of 35 g of dry soil, followed by varying amounts of hydrophobic and unmodified sand. The measurement cylinder below the samples was used to carry out infiltration studies. (b) Graph illustrates the percentage of water retained in each of the six soil systems, measured on days 1, 2, 3, 5, 7, and 14 post irrigation with different letters on the graph indicating statistically significant differences in the measurements (*p* < 0.05).

It is important to note that each sample received consistent daily irrigation of 150 mL of water throughout the duration of 14 days. The mean WRC of the control samples were 56.00 ± 4.00, 38.67 ± 2.67, 32.00 ± 4.62, and 36.00 ± 4.00% on days 2, 3, 5, and 7, respectively. By day 14, the WRC further decreased to 17.33 ± 3.52%. The significant decline in WRC observed on day 14 in the control samples is attributed to the cumulative leaching of hydrophilic and amphiphilic organic constituents over the 14-day experimental period, particularly fulvic and humic acids.^59^ These compounds are known to promote soil aggregation through chelation of the soil particles, thereby enhancing soil texture and thus its WRC.^60^ Additionally, the sustained loss of these hydrophilic and amphiphilic organic materials leads to an increase in the relative hydrophobicity of the soil matrix, reducing its affinity toward water, resulting in lower WRC.^61^

In addition, samples containing 17.70, 44.24, 88.50, and 177.00 mg/cm^2^ of hydrophobic sand exhibited a gradual decline in the WRC over time. The sample containing 796.46 mg/cm^2^ of hydrophobic sand exhibited complete inhibition of infiltration for the first 3 days. This was followed by a WRC of 105.33 ± 3.52% on day 5, after which a gradual decline in WRC was observed. By day 14, the WRC values across all six test samples converged, showing no statistically significant differences. This trend is attributed to the sustained application of a hydraulic head on the plastron between the hydrophobic sand particles, which lead to the development of sufficient capillary pathways in all samples to facilitate the drainage of excess water after the saturation of the soil.

### 3.3 Water Infiltration Kinetics through Soil Systems

The primary objective of utilizing a hydrophobic sand bed is to delay water infiltration through the soil profile. This approach is particularly critical during irrigation in arid and semiarid regions, where sandy-textured soils with low WRC are prevalent, such as in parts of Central Europe, Asia, and South America.^62^ By slowing the downward water flux through the soil layers, these strategies aim to maintain a higher water potential around plant roots and aid in efficient root uptake. If the process of water infiltration is delayed, the mucilage around the roots is saturated and helps maintain the integrity of the rhizosphere and thus ensures a positive water potential around the roots for longer periods of time.^63^

The water infiltration kinetics for days 1, 7, and 14 are presented in Figure 5. When the curves comparing water infiltration through the samples over time were compared, they exhibit characteristics of both first- and zeroth-order kinetics. Hence, the infiltration behavior was modeled using the following differential equation

$$\theta \left(t\right)=\theta_{0}\left(1-e^{-t/\tau }\right)+\alpha t$$

where θ represents the percentage of infiltrated water volume relative to the total irrigated volume (of 150 mL) to each sample, τ is the characteristic time constant (first-order rate parameter), α is the zero-order rate constant, and θ_0_ represents the total volume of water infiltrated from the sample at steady state. The first term in the fitting model describes the initial, transient phase of water infiltration. This rapid infiltration is primarily driven by capillary forces and the soil’s inherent sorptivity, which together facilitate the swift downward flow of the irrigated water through the interstitial voids between the granular soil particles. The second term represents the quasi-steady-state infiltration rate that reflects infiltration predominantly under the influence of gravity, once the interstitial gaps within the soil have become saturated.^64^ Nonlinear regression fitting of the infiltration data (θ vs time) for all tested substrates on days 1, 7, and 14 was performed using OriginPro 2021. A custom fitting function, based on the equation above, was implemented to extract the kinetic parameters τ, α, and θ_0_ for each condition. The fitting parameters for each sample across the 3 days of study (i.e., day 1, day 7, and day 14) are shown in Table 2. The goodness-of-fit for the chosen model is further substantiated by the adjusted *R* ^2^ values, presented in the last row of Table 2. These values consistently exceeded 0.96, indicating a strong agreement between the experimental data and the chosen model.

**Figure 5:**
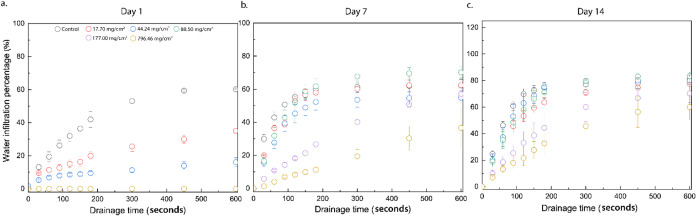
Three graphs representing cumulative water infiltration over the first 600 s (10 min) of infiltration for six distinct soil samples on (a) day 1, (b) day 7, and (c) day 14 of the experiment post irrigation with 150 mL of water.

**Table 2:** Fitting Parameters Derived from Applying a Combined First-Order and Zero-Order Kinetic Model to the Observed Cumulative Infiltration Volume during the Initial 10 Min of Water Infiltration across All of the Six Samples (Sample Names Represent the Areal Density of the Hydrophobic Sand Bed)

sample name	τ (s)	θ_0_	α (s^–1^)	adj *R* ^2^
Day 1				
control	133.66 ± 12.66	61.64 ± 2.5	(−7.33 ± 3.33) × 10^05^	0.99
17.70 mg/cm^2^	565.75 ± 93.87	42.35 ± 3.95	(3.04 ± 0.72) × 10^04^	0.99
44.24 mg/cm^2^	330.74 ± 110.09	12.32 ± 2.3	(1.11 ± 0.52) × 10^03^	0.97
Day 7				
control	57.61 ± 0.85	53.88 ± 0.47	(2.61 ± 0.15) × 10^03^	0.99
17.70 mg/cm^2^	73.67 ± 10.83	63.89 ± 2.83	(−2.88 ± 6.89) × 10^04^	0.99
44.24 mg/cm^2^	67.79 ± 4.36	62.04 ± 2.15	(1.65 ± 0.59) × 10^03^	0.99
88.50 mg/cm^2^	82.56 ± 2.81	77.37 ± 1.35	(−1.13 ± 0.24) × 10^03^	0.99
177.00 mg/cm^2^	484.204 ± 131.99	86.36 ± 13.76	(1.44 ± 5.42) × 10^05^	0.98
796.46 mg/cm^2^	1243.36 ± 408.41	91.6 ± 9.1	(1.59 ± 9.11) × 10^06^	0.96
Day 14				
control	56.94 ± 3.07	93.07 ± 3.09	(2.50 ± 6.62) × 10^03^	0.99
17.70 mg/cm^2^	110.34 ± 7.82	75.11 ± 2.37	(2.92 ± 2.94) × 10^04^	0.99
44.24 mg/cm^2^	63.2 ± 4.13	99.74 ± 5.19	(−3.06 ± 0.83) × 10^03^	0.99
88.50 mg/cm^2^	95.08 ± 2.09	88.97 ± 0.84	(−6.38 ± 0.01) × 10^00^	0.99
177.00 mg/cm^2^	200.64 ± 8.46	75.44 ± 0.96	(−5.58 ± 0.01) × 10^00^	0.99
796.46 mg/cm^2^	310.44 ± 25.31	70.77 ± 2.81	(2.35 ± 0.01) × 10^00^	0.99

From the nonlinear fitting of the infiltration data, the characteristic time constant τ can be interpreted as an indicator of the first-order rate at which water infiltrates through the soil–sand layered system. Higher the value of τ, the slower the infiltration. On day 1, the control sample (containing unmodified sand and soil) exhibited a τ value of 133.66 ± 12.66 s, reflecting relatively rapid infiltration dynamics. However, with the incorporation of hydrophobic sand at an areal density of 17.70 mg/cm^2^ corresponding to the minimum amount required to form a monolayer at the bottom of the model substrate simulated agricultural conditions, a substantial increase in τ was observed, over 4 times relative to the control. Moreover, the total infiltrated volume at equilibrium, denoted as θ_0_, decreased by approximately 1.5 times in comparison to the control.

Notably, at an areal density of 88.50 mg/cm^2^, the time constant was approximately 2.5 times higher than that of the control. Although this increase in τ is less than expected, the relative increase in the τ when 17.70 mg/cm^2^ of hydrophobic sand was utilized was higher than the samples with 88.50 mg/cm^2^ of hydrophobic sand (potentially due to fitting uncertainties); the corresponding θ_0_ value showed a significant 5-fold reduction, suggesting a stronger barrier to infiltration. For samples with an areal density of hydrophobic sand ≥88.50 mg/cm^2^, no infiltration was observed on day 1, signifying that an areal density of 88.50 mg/cm^2^ was sufficient to counter the pressure exerted by the hydraulic head (caused by 150 mL of water) on the freshly made model samples under gravity-driven flow conditions.

By days 7 and 14, all tested samples exhibited measurable water infiltration, indicating a time-dependent degradation of the hydrophobic barrier. Comparing the control samples at day 7 and day 14 to day 1, a decrease in τ by more than a factor of 2 on both the days was observed. On day 7, the control and the samples with lower areal densities of hydrophobic sand (17.70, 44.24, and 88.50 mg/cm^2^) showed insignificant differences in infiltration kinetics. The samples with higher areal densities, i.e., 177.00 and 796.46 mg/cm^2^, continued to demonstrate significantly delayed infiltration. Specifically, the characteristic time constants τ for these samples were approximately 8.5- and 22.0-fold higher than the control on day 7, respectively, and 3.5 and 5.5 times greater than the control on day 14. Even after 14 days of continuous irrigation: oversaturating with 150 mL of water, the samples with 796.46 mg/cm^2^ areal density of hydrophobic sand showed 1.3 times lower θ_0_ when compared to the control. Significant fluctuations were observed in the fitting parameters, particularly under slow infiltration conditions. Notably, this variability was evident in the τ values for 44.24 mg/cm^2^ samples on day 1, for both 177.00 and 796.46 mg/cm^2^ samples on day 7, and for the 796.46 mg/cm^2^ sample on day 14. This phenomenon can likely be attributed to the heterogeneous granular arrangement and packing of the soil and hydrophobic sand beds, resulting in variance in the stability of the plastron, which subsequently leads to dissimilar capillary-pathway-assisted flow profiles.

To provide a simplified understanding of the infiltration process within each of the six test samples under simulated agricultural conditions, we categorized the observed infiltration behavior into three distinct classifications: rapid infiltration (infiltration rates not significantly different from the control samples), delayed infiltration (infiltration rates significantly slower than the control), and no infiltration. These classifications are summarized in Table 3. Furthermore, to visually demonstrate the infiltration patterns, we have included three videos (Supporting Information Videos 1–3) illustrating infiltration under varying areal concentrations of hydrophobic sand (refer to the captions below each video for detailed information about the samples demonstrated). Additionally, snapshots at three specific time points *t* = 1, 5, and 10 min are provided in Figures S2–S4 to further illustrate the progression of infiltration.

**Table 3:** Classification of All of the Experimental Setups into 3 Infiltration Conditions, Namely, Rapid Infiltration, Delayed Infiltration, and No Infiltration

	rapid infiltration	delayed infiltration	no infiltration
day 1	control	17.70, 44.24 mg/cm^2^	88.50, 177.00, 796.46 mg/cm^2^
day 7	control, 17.70, 44.24, 88.50 mg/cm^2^	177.00, 796.46 mg/cm^2^	
day 14	control, 17.70, 44.24, 88.50 mg/cm^2^	177.00, 796.46 mg/cm^2^	

The observed infiltration kinetics can be divided into two parts: first, the infiltration kinetics in the freshly prepared samples on day 1 as a function of the varying thickness (areal density) of the hydrophobic sand particles; and second, the depletion of the plastron (present within the interstitial spaces between the layers of the hydrophobic sand) under daily irrigation, i.e., oversaturation of the soil systems. This depletion resulted in accelerated infiltration observed across all six samples on day 7, with a further increase in acceleration on day 14, compared with their respective previous time points.

To understand the infiltration kinetics observed on day 1, a comparison can be drawn between water transport through the hydrophobic sand layer, as is the case in this paper and water transport through porous hydrophobic membranes, which is thoroughly discussed in the literature.^65^ As described by Chamani et al., the liquid entry pressure, minimum hydrostatic pressure required to overcome the capillary forces resisting liquid entering into the pores, is directly dependent on the volume of the trapped plastron residing within the interstitial spaces of the membrane.^66^ Hence, when the areal density of the hydrophobic sand was ≥88.50 mg/cm^2^, the capillary resistive forces offered by the trapped plastron were sufficient to withstand the applied hydrostatic pressure head applied by 150 mL of water. According to Darcy’s Law, increased flow resistance, offered by the increased plastron volume (higher areal densities of hydrophobic sand) leads to a decrease in hydraulic conductivity.^67^ Hence, we observe an increase in the characteristic time constant, τ, with increasing areal density of the hydrophobic sand, at each time point.

The accelerated water infiltration on day 7 and day 14 could be attributed to the diffusion of amphiphilic organic constituents occurring from the hydraulic head toward the hydrophobic sand layer, driven by interfacial interactions and minimization of Gibbs interfacial free energy.^68^ This phenomenon can be well understood by drawing comparisons with Ward–Tordai relation.^69^ This dynamic diffusion process involves the bidirectional transport of amphiphilic dissolved organic matter, characterized by diffusion from the overlying hydraulic head toward the hydrophobic sand layer, followed by back-diffusion from the hydrophobic sand interface into the bulk aqueous phase.^70,71^ This amphiphilic movement may facilitate the interfacial rearrangement of the hydrophobic sand layers and thus eventually destabilize and release the plastron. The loss of these air pockets potentially leads to the formation of capillary pathways, thereby enabling water to infiltrate more readily through the hydrophobic soil matrix. This phenomenon leads to progressive mixing of the dark, brown topsoil with both unmodified and hydrophobic sand layers, observed over time under daily irrigation. Refer to Supporting Videos 1 and 3 for a visual comparison.

Furthermore, a detailed examination of Video 1, specifically the no-infiltration condition, reveals evidence of saltation behavior: characterized by vertical motion of the sediments (here in this case sand particles) due to the resistance offered to the water flow.^72,73^ This phenomenon appears to be driven by the hydraulic pressure exerted by the water column, in conjunction with the release plastron from within the interstitial spaces of the hydrophobic sand layer. The repeated upward ejection and subsequent gravitational settling of sand particles contribute to the disruption of the interstitial air pockets, thus facilitating rapid water infiltration.

### 3.4 CIELAB Analysis on Soil and Infiltrated Water

As previously reported, soil is composed of hydrophilic and amphiphilic components along with various forms of organic matter. The hydrophilic and amphiphilic fractions of soil organic matter are primarily represented by fulvic acid and humic acid, respectively, which contribute to improved soil texture and water retention capacity.^74^ In addition, they play several vital roles in soil chemistry, enhancement of soil buffering capacity, and increased bioavailability of essential nutrients such as nitrogen and phosphorus.^75,76^ Previous literature has correlated the soil nutrient contents, such as C, N, and Fe, to the CIELAB color parameters.^77^ In addition, researchers in the past have shown that the yellowish-brown coloration imparted to the infiltrated water is due to the dissolution of hydrophilic and amphiphilic organic matter (predominantly fulvic and humic acid).^78^ Hence, colorimetric analysis was performed by using the CIELAB color space on both the infiltrated water and the soil samples across six different experimental setups.

The following observations can be made from the data presented in Figure 6: the L*, hue angle, and Chroma values obtained from colorimetric analysis of the infiltrated water and dried soil samples.

**Figure 6:**
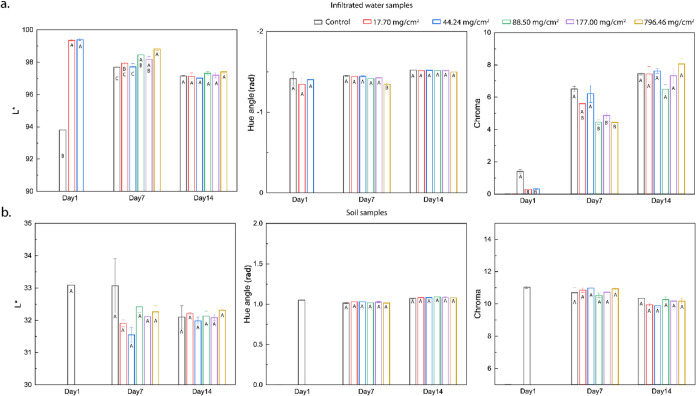
CIELAB colorimetric parameters of six soil samples, presented using L*, hue angle, and chroma of (a) water infiltrated through the samples post irrigation and (b) corresponding soil samples after drying. The statistical distinction in the data is represented by the letters (*p* < 0.05).

First, the CIELAB color space parameters of the soil samples did not exhibit any statistically significant variations across the three observation days (day 1, day 7, and day 14). Consistent with previous research correlating soil nutritional content with CIELAB parameters, this suggests that the soil nutrient composition remained largely unchanged across all samples, despite variations in the proportions of hydrophobic and unmodified sand and the change in the total water infiltrated through each sample (over all 14 days). This insignificant change is likely attributable to the relatively short duration of the experiment compared with the field conditions (where continuous irrigation followed by infiltration takes place over the span of several months), which may have been insufficient to elicit detectable differences in the soil nutrient content.

As no water infiltration was observed on day 1 when the areal density of hydrophobic sand was ≥88.5 mg/cm^2^, the CIELAB color parameters were not reported. Analysis of other infiltrated water samples revealed no statistical difference in the hue angle, indicating that the solute composition of dissolved organic matter remained consistent across samples, with only variations in concentration. However, on day 1, the control water exhibited a significantly lower L* value and higher chroma. This observation can be attributed to the infiltration of some of the unmodified sand particles (with a granular size less than the mesh size of the cheesecloth), as evidenced by hazy looking infiltrated water in Video 1.

When the L* values of the infiltrated water samples were observed, a statistically significant difference in brightness/darkness was observed only on day 7, with varying amounts of hydrophobic sand. The L* values consistently increased with increasing amounts of hydrophobic sand. The highest L* value was observed at hydrophobic sand concentration of 796.46 mg/cm^2^ (denoted by the letter A), followed by 88.50 and 177.00 mg/cm^2^ (grouped as AB). Conversely, on day 7, the chroma values decreased with increasing hydrophobic sand content with areal concentrations of 88.50, 177.00, and 796.46 mg/cm^2^ (grouped in B), showing significantly lower chroma values in comparison to the control samples.

The interaction between the hydrophobic sand and the amphiphilic dissolved organic matter is predominantly hydrophobic. Hence, the hydrophobic interactions between the organic matter and the hydrophobic sand determine the extent of dissolved organic matter in the infiltrated water. According to Langmuir’s adsorption isotherm, the equilibrium adsorption of amphiphilic molecules (given that water infiltration continued for 1 h) is directly dependent on the maximum adsorption capacity (*q*_m_), which, in turn, is proportional to the amount of hydrophobic sand granules in each sample.^79,80^ Therefore, during water infiltration, a fraction of the amphiphilic dissolved organic matter is adsorbed onto the hydrophobic sand granules, which is proportional to the quantity of hydrophobic sand in the system. Hence, an increase in L* and a decrease in Chroma were observed on day 7 with increasing areal densities of the hydrophobic sand.

When the color on the infiltrated water samples on day 14 was analyzed, no statistical distinction difference in L*, hue, and chroma was observed. As a consequence of the preceding 13 days of irrigation, subsequent infiltration saturated the adsorption sites on the hydrophobic sand, i.e., *q*_e_ (equilibrium fraction binding capacity on the hydrophobic sand) is equal to *q*_max_ (maximum binding capacity).^81^ Hence, no distinction in the infiltrated samples was observed.

To further mitigate the loss of amphiphilic dissolved soil organic matter to infiltrated water even in prolonged durations of irrigation, this approach of utilizing the hydrophobic sand bed, designed to impede rapid water infiltration, should be combined with other strategies like timely replenishment of the top layers of the soil and use of soil microbiota to achieve a synergistic effect.^82^ Additionally, future research should investigate methods to enhance the thermal stability of the plastron within soil systems.

Given the observed differences in dissolved organic matter in the infiltrated water on day 7, the relative comparison of dissolved organic matter was quantified by measuring the percentage decrease in absorbance at 287 nm using UV–visible spectroscopy compared to control samples (Table 4).

**Table 4:** Percentage Decrease in Dissolved Organic Matter in Infiltrated Water on Day 7, Relative to Control Samples, with the Letters in the Brackets Representing Statistical Distinction (*p* < 0.05)

sample name	decrease in the dissolved organic matter (%)
17.70 mg/cm^2^	13.68 ± 2.65 (B)
44.24 mg/cm^2^	16.61 ± 4.68 (B)
88.50 mg/cm^2^	15.55 ± 1.70 (B)
177.00 mg/cm^2^	25.67 ± 3.02 (A)
796.46 mg/cm^2^	23.96 ± 3.85 (A)

Statistical analysis identified two distinct groups. Samples with hydrophobic sand bed concentrations of 17.70, 44.24, and 88.50 mg/cm^2^ were grouped into Class B and samples with concentrations of 177.00 and 796.46 mg/cm^2^ were classified into Class A, demonstrating approximately 25% lower dissolved organic matter content in the infiltrate compared to control when higher areal densities (177.00 and 796.46 mg/cm^2^) of hydrophobic sand bed were utilized, even after 7 days.

### 3.5 Impact on Plant Growth

Previous sections have focused on characterizing key soil parameters, including the incorporation of hydrophobic sand to enhance water retention, delay infiltration, and its effects on amphiphilic organic matter present in the infiltrated water. To provide a more comprehensive understanding of our novel soil amendment concept and to establish a foundation for future research, we investigated the impact of the hydrophobic sand bed on plant growth. Of particular interest was the growth of seedlings in the two sample groups following the termination of irrigation. As illustrated in Figure 7, both on day 4 and day 10 post seizure of irrigation, a direct positive correlation was observed between enhanced water retention in the sample containing hydrophobic sand and a significant increase in the growth of tomato seedlings. Specifically, seedlings cultivated with the hydrophobic sand bed exhibited approximately twice the growth stature compared with those grown in unmodified soil.

**Figure 7:**
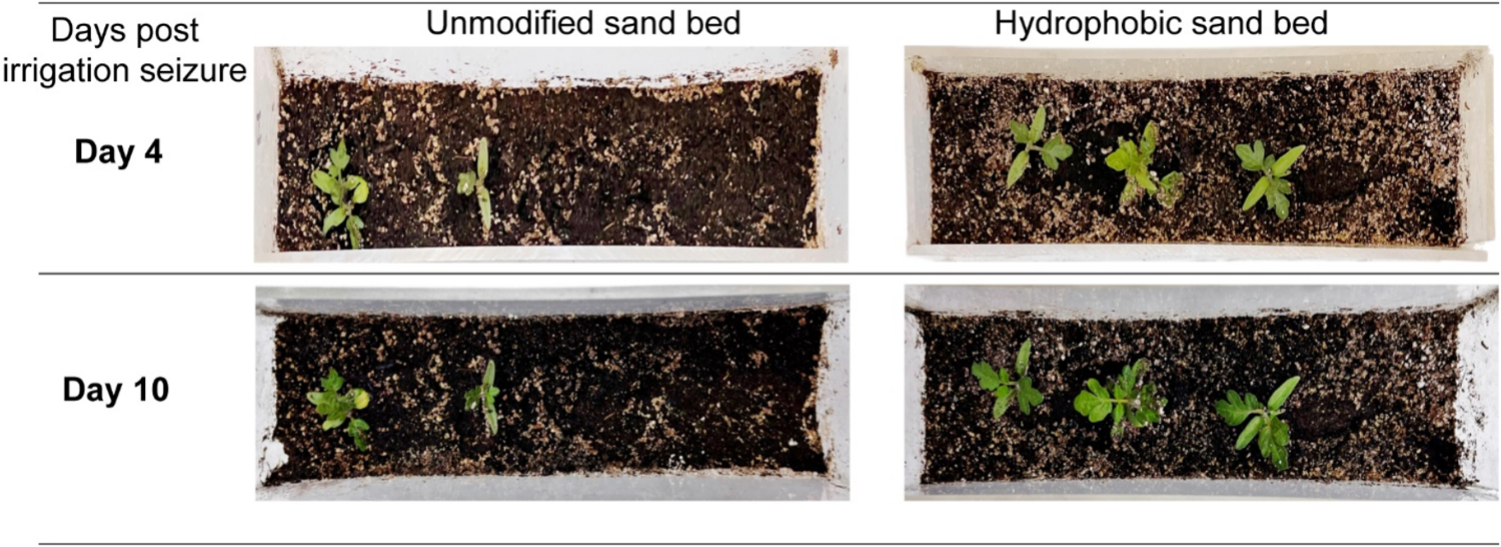
Digital images of two model samples simulating seedling growth conditions under differing sand beds: one with an unmodified sand bed and the other with a hydrophobic sand bed. Images were captured 4 and 10 days following irrigation cessation to evaluate postseizure plant response and substrate effects on moisture retention and seedling viability.

In the sample with a hydrophobic sand bed, greater water potential in the rhizosphere, resulting from enhanced water retention, facilitates sustained plant growth even in the absence of irrigation. This becomes particularly critical in hot arid regions: parts of Central Asia, Australia, and North Africa with prolonged desert-like conditions.^83^ This preliminary experiment aimed to provide a broad overview and demonstrate that the incorporation of a hydrophobic sand bed positively impacts seedling growth under nonirrigated conditions due to its enhanced water retention capabilities. Further investigations are needed on the impact of hydrophobic soil on rhizobacteria, effect on plant growth, physiology, and resistance toward biotic stress and overall health of the plants.^84,85^

In the current experimental procedure, the hydrophobic sand was prepared ex situ, and rigorous washing ensured no residual or unreacted OTS was present before the hydrophobic sand bed’s preparation. OTS is highly reactive to moisture, undergoing hydrolysis to form hydrochloric acid (HCl).^86^ The resulting HCl could have negative impacts on agricultural soil and groundwater.^87^ Therefore, for potential agricultural-scale applications, advanced techniques for in situ hydrophobization of sand beds need to be explored. These include targeted delivery of OTS or the addition of acid neutralizers such as calcium and magnesium carbonate, or phosphate rocks, during the conditioning of sand beds prior to cultivation.^86,88^

## 4 Conclusions

This research demonstrates the efficacy of subrhizosphere organosilanization, specifically using octadecyltrichlorosilane modification of silica sand, as a targeted strategy to engineer hydraulic barriers for agricultural water conservation. Spectroscopic analysis (ATR-FTIR) confirmed successful covalent functionalization, evidenced by characteristic C–H vibrational modes and shifts in Si–O–Si stretching frequencies, indicative of the Si–O–Si bond formation between the OTS and the sand substrate. The resultant hydrophobic sand exhibited a high static water contact angle (133.0 ± 1.0°) and maintained robust nonwetting behavior (SCA > 123°, indicative of a Cassie–Baxter state) under simulated environmental stressors, including varied pH (4.0 and 9.0), dissolved soil organic matter, and UV-AB irradiation.

Systematic evaluation of water infiltration dynamics in model soil columns revealed a strong correlation between the areal density of the hydrophobic sand layer and the impedance to vertical water flux. Quantitative analysis using a combined first- and zero-order kinetic model showed that increasing the areal density significantly increased the characteristic infiltration time constant (τ). Notably, an areal density of 796.5 mg/cm^2^ resulted in a τ value approximately 5.5 times greater than the control system even after 14 days of sustained daily irrigation under oversaturation conditions, highlighting the durability of the induced hydraulic impedance attributed to metastable plastron entrapment. While initial complete flow inhibition was observed for densities of ≥88.50 mg/cm^2^, gradual degradation occurred under prolonged hydraulic head, likely mediated by amphiphilic solute interactions at the interface facilitating plastron destabilization and capillary-pathway formation, a phenomenon analogous to Ward–Tordai interfacial dynamics.

Furthermore, CIELAB and UV–vis analyses of leachate provided indirect evidence for enhanced retention of dissolved organic matter within the soil profile overlying the hydrophobic barrier, indicated by significantly higher L* (brightness) and lower Chroma values in leachate from systems with higher hydrophobic sand densities, particularly at intermediate time points (day 7). This suggests a secondary benefit of mitigating nutrient loss via leaching. Preliminary pot experiments corroborated the primary hypothesis, demonstrating significantly enhanced tomato seedling growth and survival postirrigation cessation in systems incorporating the hydrophobic layer, directly attributable to superior water retention within the effective root zone.

In conclusion, subsoil hydrophobization via in situ applicable organosilane treatment presents a scientifically sound and potentially impactful approach for enhancing agricultural water use efficiency. By precisely controlling pore-scale wetting properties and inducing stable plastron formation, this method effectively slows deep percolation, increases the water residence time in the rhizosphere, and shows potential for conserving soil organic constituents. While these laboratory-scale findings are promising, future research must focus on optimizing treatment protocols for long-term plastron stability, validating performance across diverse soil types and field conditions, conducting thorough technoeconomic analyses for scalability, and assessing potential long-term ecological impacts.

## Supplementary Material









## Notes

Complete FTIR spectra to show the successful surface modification of silica sand (PDF)
Video demonstration of water infiltration on day 1 through control, 17.70 and 796.46 mg/cm^2^ samples captured immediately after irrigation of the samples (Video S1) (MP4)
Video demonstration of water infiltration on day 7 through control, 177.00 and 796.46 mg/cm^2^ samples captured immediately after irrigation of the samples (Video S2) (MP4)
Video demonstration of water infiltration on day 14 through control and 796.46 mg/cm^2^ samples captured immediately after irrigation of the samples (Video S3) (MP4)
