# Supplementary material for: Organosilanized Hydrophobic Sand for Drought Resilience: Reducing Water Percolation and Enhancing Crop Growth Conditions
Source: ACS Omega. 2025 Aug 14;10(33):37583–96. doi: 10.1021/acsomega.5c03952 (PMC12392176; doi:10.1021/acsomega.5c03952)
Supplement: Supplementary file 1 [file ao5c03952_si_001.pdf]

Supplementary Information to

Organosilanized Hydrophobic Sand for Drought Resilience: Reducing Water Percolation and Enhancing Crop Growth Conditions

Yashwanth Arcot[a], Ramya Srinivas[a], Minchen Mu[a], Mahshad Maghoumi[b], Luis Cisneros-Zevallos[b], and Mustafa E. S. Akbulut[a, c*]

[a] Artie McFerrin Department of Chemical Engineering, Texas A&M University, College Station, TX 77843, USA
[b] Department of Horticultural Sciences, Texas A&M University, College Station, TX 77843, USA
[c] Department of Materials Science and Engineering, Texas A&M University, College Station, TX 77843, USA

[*]Corresponding author: E-mail address: makbulut@tamu.edu.

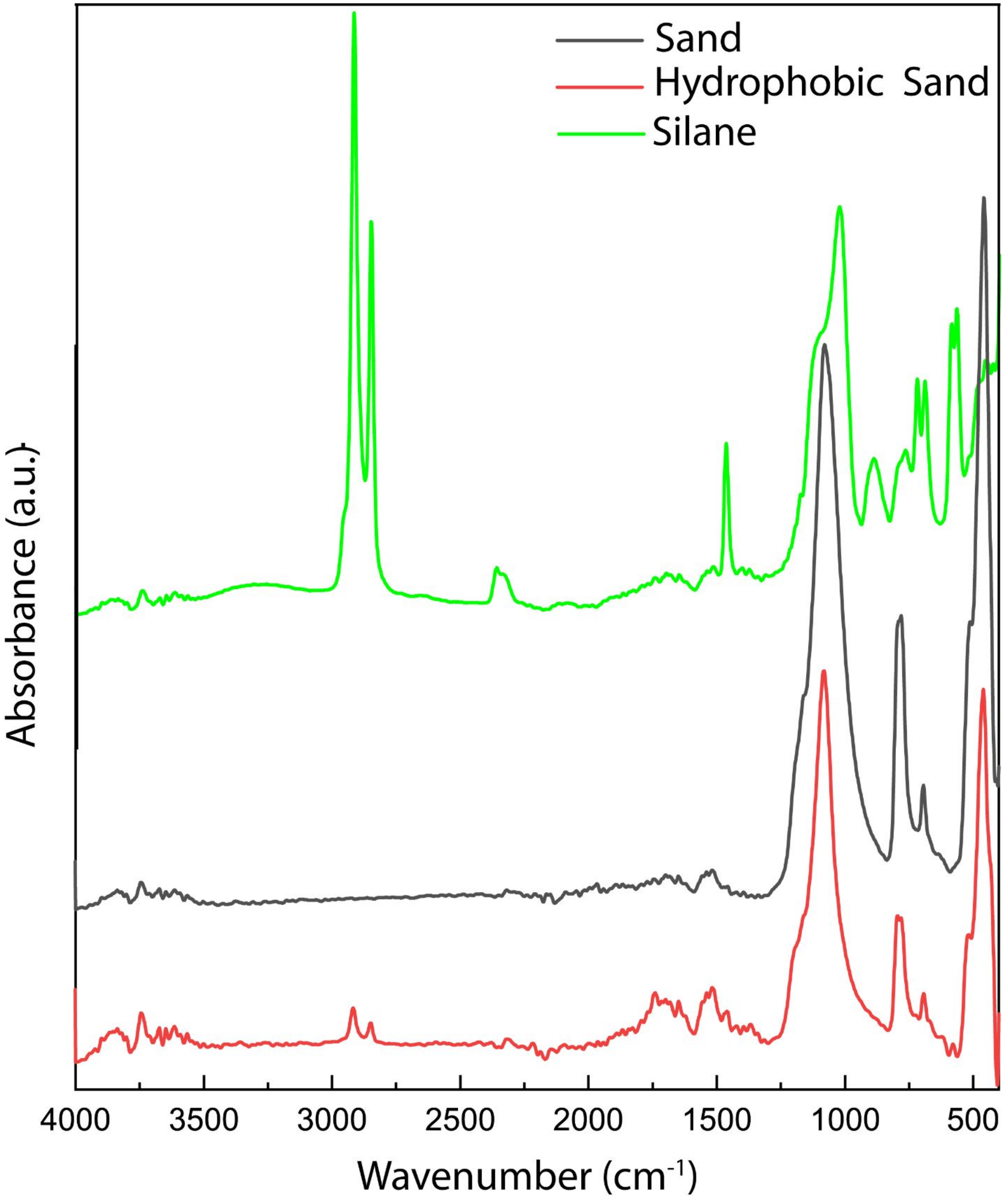

Figure S1 Complete ATR-FTIR spectra (4000-400 cm^{-1}) illustrating the surface functionalization of silica sand.

Water infiltration - snapshots

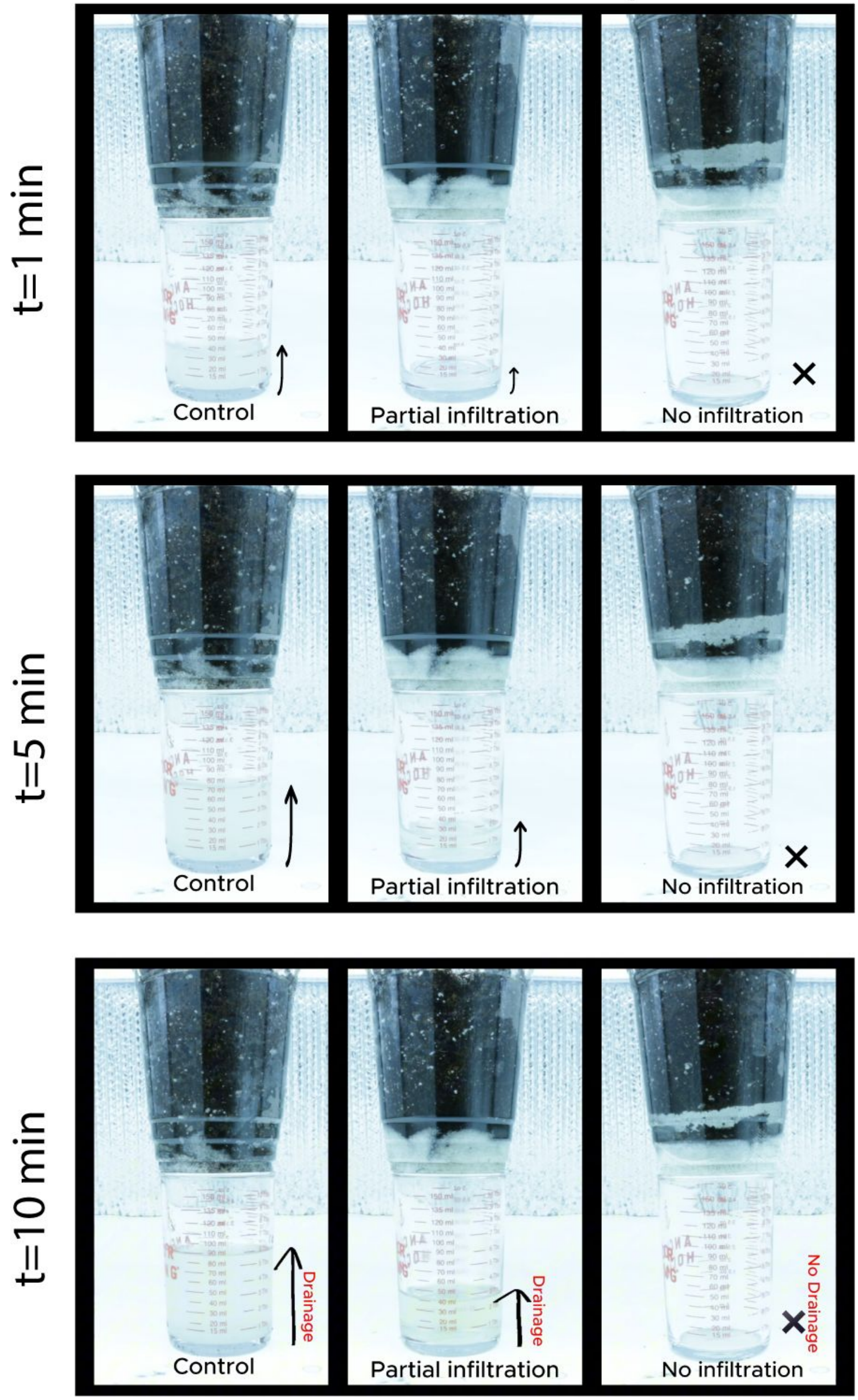

Figure S2 Snapshots captured from water infiltration video recordings on day 1 on control, 17.70 mg/cm^2 and 796.46 mg/cm^2 samples. Snapshots were captured at 1, 5, and 10 minutes timepoints after the initiation of infiltration. Arrows indicate the position of the water level in the measuring cylinder.

Water infiltration - snapshots

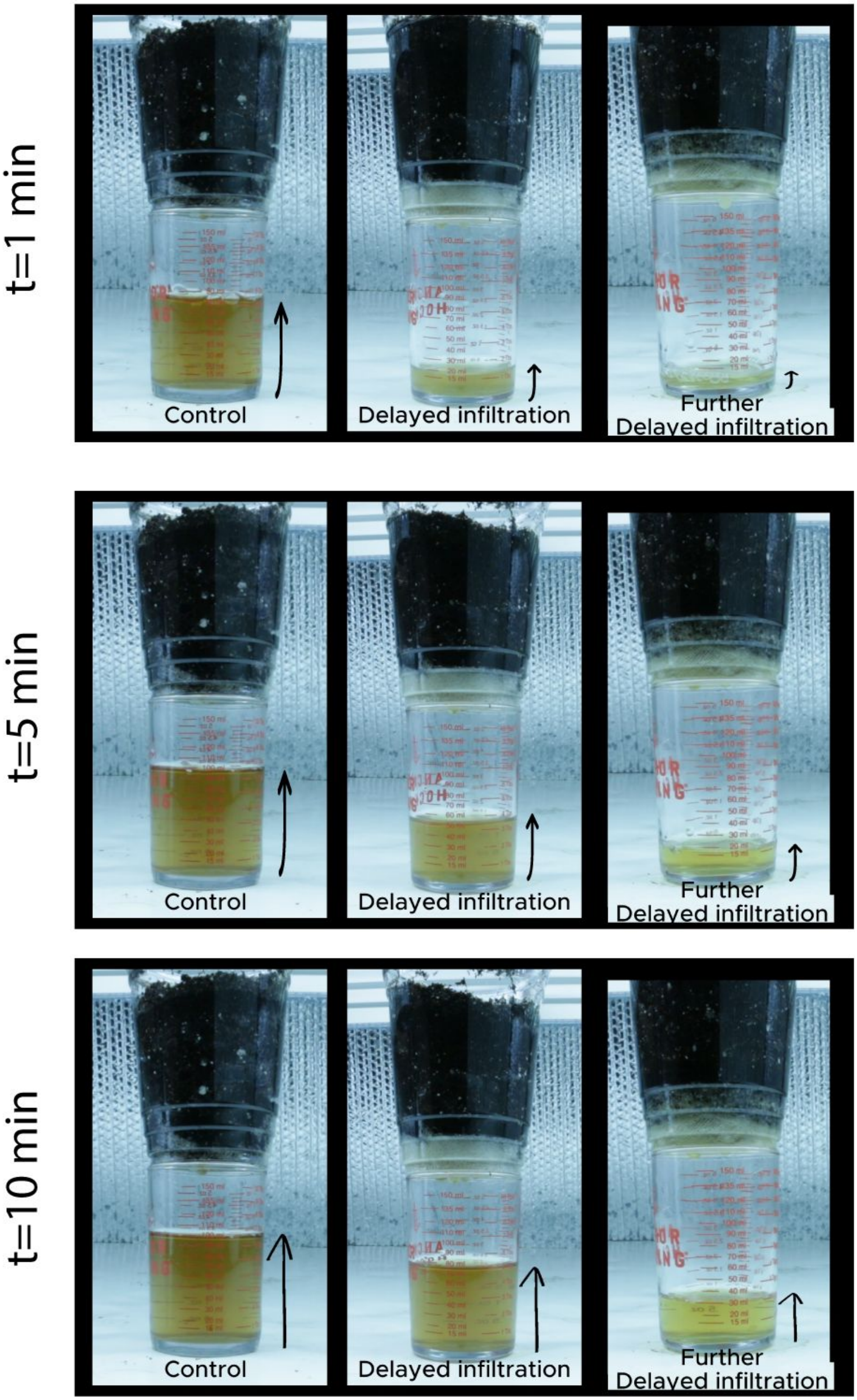

Figure S3 Snapshots captured from water infiltration video recordings on day 7 on control, 177.00 mg/cm^2 and 796.46 mg/cm^2 samples. Snapshots were captured at 1, 5, and 10 minutes timepoints after the initiation of infiltration. Arrows indicate the position of the water level in the measuring cylinder.

Water infiltration - snapshots

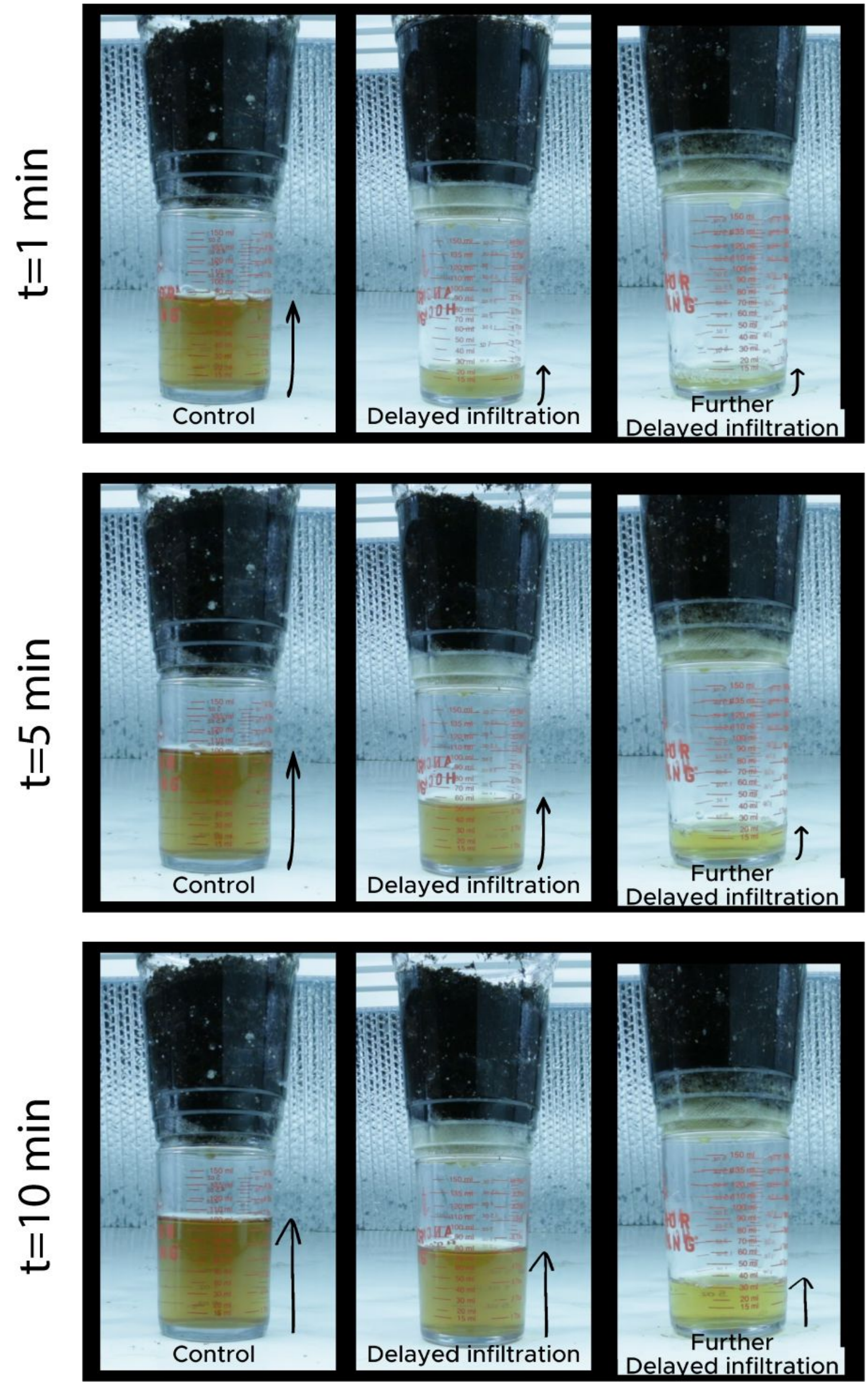

Figure S4 Snapshots captured from water infiltration video recordings on day 14 on control, and 796.46 mg/cm^2 samples. Snapshots were captured at 1, 5, and 10 minutes timepoints after the initiation of infiltration. Arrows indicate the position of the water level in the measuring cylinder.

V1.mp4

Video 1 Video demonstration of water infiltration on day 1 though control, 17.70 mg/cm^2 and 796.46 mg/cm^2 samples captured immediately after irrigation of the samples.

V2.mp4

Video 2 Video demonstration of water infiltration on day 7 though control, 177.00 mg/cm^2 and 796.46 mg/cm^2 samples captured immediately after irrigation of the samples.

V3.mp4

Video 3 Video demonstration of water infiltration on day 14 though control and 796.46 mg/cm^2 samples captured immediately after irrigation of the samples.
